# Accumulation of marginal zone B cells and accelerated loss of follicular dendritic cells in NF-κB p50-deficient mice

**DOI:** 10.1186/1471-2172-6-8

**Published:** 2005-04-18

**Authors:** Andrew R Ferguson, Ronald B Corley

**Affiliations:** 1 Department of Microbiology, Boston University School of Medicine, 715 Albany Street, Boston, MA 02118 USA

## Abstract

**Background:**

Marginal zone (MZ) B cells play important roles in the early phases of humoral immune responses. In addition to possessing an inherent capacity to rapidly differentiate into antibody secreting cells, MZ B cells also help to regulate the fate of both T-independent and T-dependent blood-borne antigens in the spleen. For T-dependent antigens, MZ B cells bind IgM-antigen complexes in a complement-dependent manner. Once MZ B cells bind IgM-containing immune complexes (IgM-IC), they transport them into B cell follicles for deposition onto follicular dendritic cells (FDCs), an important component of secreted IgM's ability to enhance adaptive immune responses. To further define the requirement for MZ B cells in IgM-IC deposition, mice deficient in the NF-κB protein p50, which have been reported to lack MZ B cells, were analyzed for their ability to trap IgM-IC onto FDCs.

**Results:**

Mice (2 months of age) deficient in p50 (p50^-/-^) had small numbers of MZ B cells, as determined by cell surface phenotype and localization in the splenic MZ. These cells bound high levels of IgM-IC both *in vivo *and *in vitro*. Subsequent to the binding of IgM-IC by the MZ B cells in p50^-/- ^mice, small amounts of IgM-IC were found localized on FDCs, suggesting that the MZ B cells retained their ability to transport these complexes into splenic follicles. Strikingly, MZ B cells accumulated with age in p50^-/- ^mice. By 6 months of age, p50^-/- ^mice contained normal numbers of these cells as defined by CD21/CD23 profile and high level expression of CD1d, CD9, and IgM, and by their positioning around the marginal sinus. However, FDCs from these older p50^-/- ^mice exhibited a reduced capacity to trap IgM-IC and retain complement components.

**Conclusion:**

These results demonstrate that while the p50 component of the NF-κB transcription complex plays an important role in the early development of MZ B cells, MZ B cells can develop and accumulate in mice lacking this protein. These results highlight the interface between genetic deficiencies and age, and suggest that different transcription factors may play distinct roles in the development and maintenance of cell populations at different ages.

## Background

Secreted IgM antibodies play important roles in the initial phases of humoral immune responses. Unlike other antibody isotypes, secreted IgM uniquely functions as an adjuvant, promoting primary antibody responses to sub-immunogenic doses of T-dependent antigens [1-7]. The adjuvanticity of IgM is completely dependent on an intact complement system [7-9]. While mice deficient in secreted IgM make protective antibody responses to pathogens, these responses are delayed compared to the immune response of IgM sufficient mice [10-13] Interestingly, pathogens more readily disseminate into vital organs in secreted IgM-deficient mice, rather than concentrate them into secondary lymphoid organs [14]. This suggests that an important role for IgM is to serve as an arbiter of antigen localization, which may contribute to its adjuvant function(s).

To investigate how IgM affects the localization of foreign antigen, a system was developed to follow the fate of IgM-containing immune complexes (IgM-IC) following their intravenous (i.v.) administration [7,15] IgM-IC first localize to the splenic marginal zone (MZ), where they are phagocytized by MZ macrophages and are also bound to MZ B cells. Binding of IgM-IC to MZ B cells is strictly complement dependent. The MZ B cells subsequently transport the IgM-IC into the B cell follicles where they are deposited onto follicular dendritic cells (FDCs) [15]. The important role of MZ B cells in this process was established by monitoring IgM-IC deposition onto FDCs in mice that lacked MZ B cells, either as a result of a genetic lesion (CD19-deficient mice) that inhibited MZ B cell survival, or by their depletion using pertussis toxin. In these mice, little if any IgM-IC was deposited onto FDCs. In addition, adoptive transfer of B cells with bound IgM-IC into mice led to IgM-IC deposition onto FDCs. In these mice, transferred MZ B cells in close proximity to FDCs were found to have IgM-IC capped and directed toward the FDC dendrites, strongly suggesting IgM-IC was being transferred to FDCs by MZ B cells [15].

To further investigate the role of MZ B cells in IgM-IC transport, we used mice deficient in p50, a member of the NF-κB family of transcription factors [16]. These mice have been reported to lack MZ B cells, suggesting an unambiguous role for this component of the NF-κB transcription factor complex in the development of this subset of B cells [17]. However, we found that small numbers of MZ B cells are present in young p50^-/- ^mice, and these cells are capable of binding high levels of IgM-IC *in vivo *and *in vitro*. Interestingly, MZ B cells were found to accumulate with age in p50^-/- ^mice such that by 6 months a normal sized MZ B cell compartment was present. We also found that p50^-/- ^mice exhibited an accelerated loss of FDC networks, as assessed by intense CD21 staining. These data indicate that MZ B cells can develop in the complete absence of p50, and suggest that the transcriptional requirements for the development and/or survival of MZ B cells might differ in young and old mice.

## Results

### Presence of MZ B cells in p50-deficient mice

**Figure 1:**
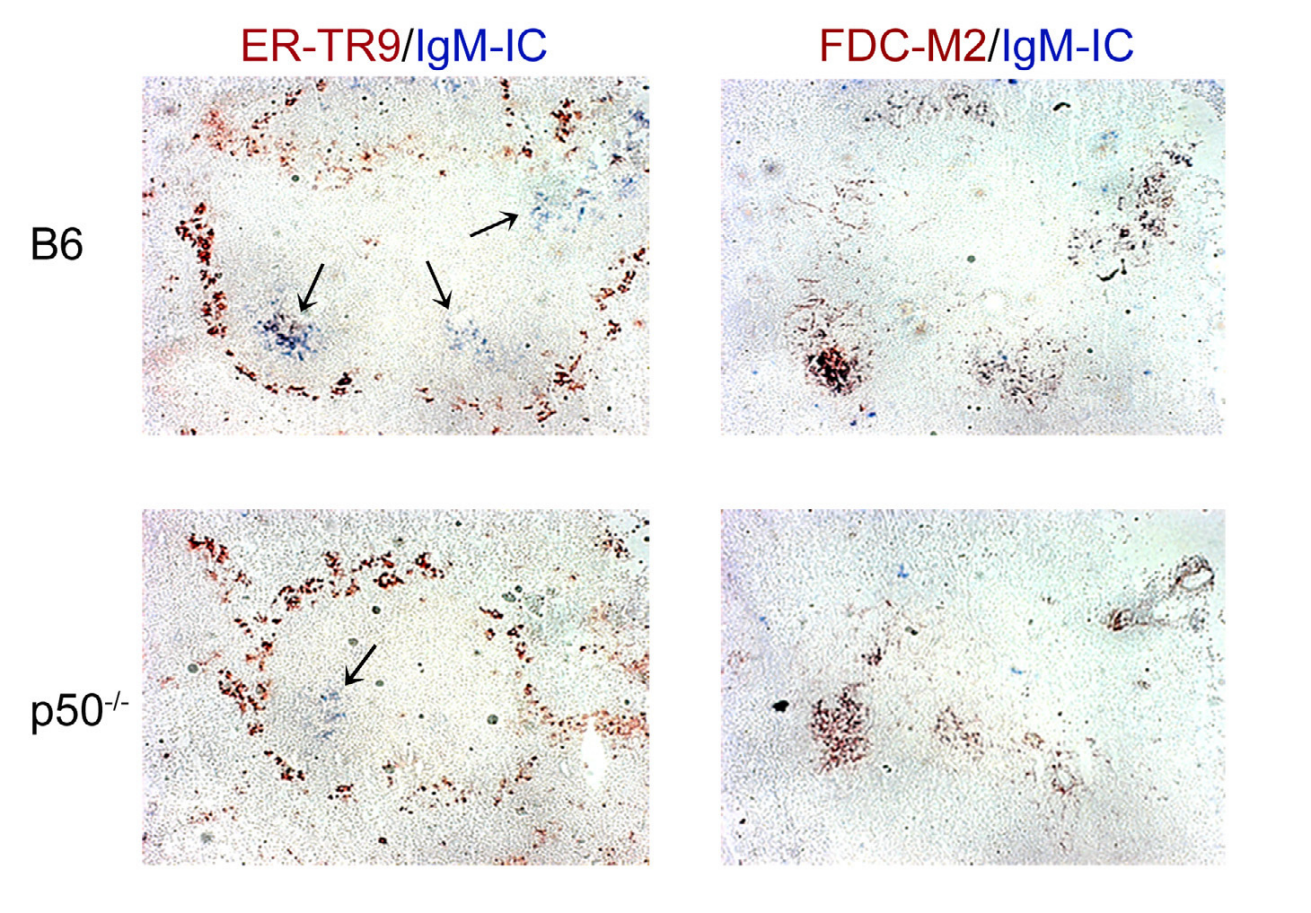
**p50^-/- ^mice trap IgM-IC less efficiently on FDCs. **Localization of IgM-IC in B6 or p50^-/- ^mice was determined by IH 16 h after injection. ER-TR9 identifies marginal zone macrophages (MZM) and FDC-M2 identifies FDCs. IgM-IC (which appear blue in this and all subsequent figures and are identified by arrows) are localized to FDCs in spleens from both groups of mice, but FDCs from B6 mice show more intense staining, indicating that more IgM-IC are trapped in these mice. All IH stains were performed on serial sections and were developed side-by-side for identical periods of time.

Two month old p50^-/- ^or control C57BL/6 (B6) mice were injected with IgM-IC and the spleens removed 16 h later. Based on the reported lack of MZ B cells in p50^-/- ^mice [17] and the requirement for MZ B cells in the transport and deposition of IgM-IC onto FDCs [15], we expected that IgM-IC would not be found localized on FDCs in the splenic follicles of these mice. However, immunohistochemistry (IH) revealed the presence of IgM-IC on FDCs in p50^-/- ^mice (Figure 1). The amount of IgM-IC localized in the spleen follicles of p50^-/- ^mice was substantially less than in control B6 mice, but none-the-less readily detectable. These results could indicate either that IgM-IC can traffic to FDCs by a previously unanticipated mechanism independent of MZ B cells, or p50^-/- ^mice may possess small numbers of MZ B cells that are still functional for transporting IgM-IC into follicles.

**Figure 2:**
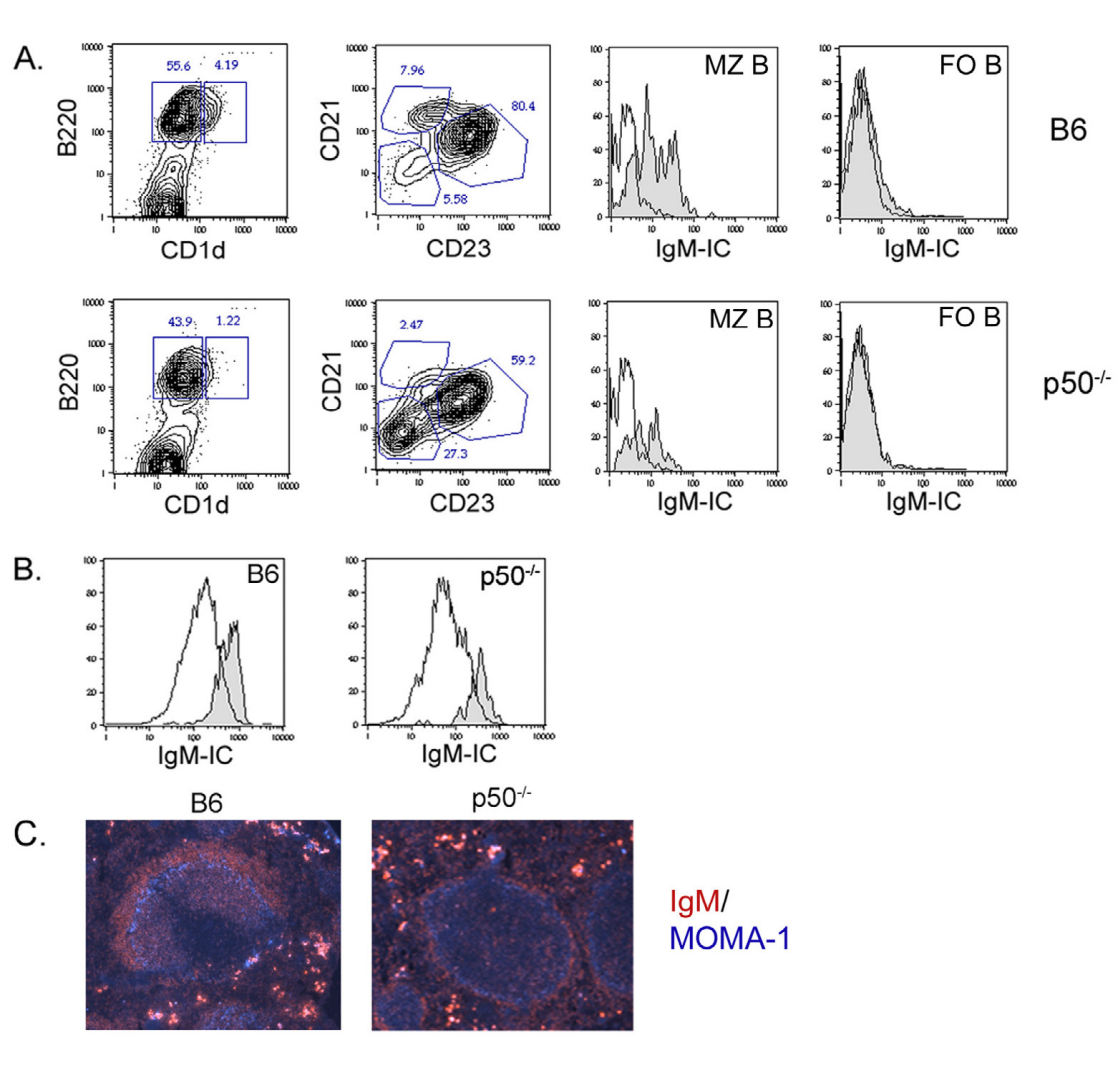
**p50^-/- ^mice contain reduced numbers of MZ B cells that bind high levels of IgM-IC A. **B cell subsets and IgM-IC binding. Splenocytes from B6 or p50^-/- ^mice were analyzed for B cell subsets by FACS using CD21/CD23 or B220/CD1d expression. Histograms on the right show binding of IgM-IC to MZ and FO B cells 1 h after i.v. injection (shaded region); clear profiles represent uninjected control mice. Although spleen cells from p50^-/- ^mice contained reduced numbers of MZ B cells, the cells present in the MZ B cell gate bind IgM-IC at high levels over background, similar to levels of binding of IgM-IC to B6 MZ B cells. **B. ***In vitro *binding assay showing binding of IgM-IC to MZ B cells (shaded region) compared with FO B cells (clear region). MZ B cells from both B6 and p50^-/- ^mice bind high levels of IgM-IC compared to FO B cells. **C. **Immunofluorescence shows the presence of IgM^hi ^cells (red) in the MZ as demarcated by MOMA-1 (marginal metallophilic macrophages, blue) staining. p50^-/- ^mice possess some IgM^+ ^cells in the MZ outside of the MOMA-1 ring, consistent with the presence of appropriately positioned MZ B cells in these mice.

To help discriminate between these alternatives, splenic B cells from p50^-/- ^mice were analyzed for the presence of MZ B cells by FACS. MZ B cells can be phenotypically distinguished from other B cell subsets in the spleen based on CD21/23 [18,19] and B220/CD1d [20,21]. profiles. As shown in Figure 2, MZ B cells were, as expected [17], severely reduced in p50^-/- ^mice. However, small numbers of B cells fell within the CD21^hi^/CD23^lo ^and B220^+^/CD1d^hi ^gates, at ~10% of the frequency found in the B cell compartment of control B6 mice. To determine if these MZ B cells could bind high levels of IgM-IC, spleen cells from mice that had been injected 1 h earlier were isolated and the presence of IgM-IC on B cell subsets was determined by FACS. When IgM-IC were injected into B6 mice, the complexes were found associated with MZ B cells at high levels (Figure 2A), as expected from their high level expression of the complement receptor CR2 (CD21) and localization at the marginal sinus, which facilitates exposure to blood-borne IgM-IC [15]. In contrast, little if any of the IgM-IC were present on follicular (FO) B cells. In p50^-/- ^mice, there was discernable binding of IgM-IC to MZ B cells (as defined by a shift in the FACS profile) while, as seen in B6 mice, IgM-IC did not bind to FO B cells (Figure 2A). This suggests that the cells in the CD21^hi ^gate in p50^-/- ^mice were authentic MZ B cells. To confirm this, the capacity of these cells to bind IgM-IC was determined using *in vitro *binding assays (Figure 2B). When p50^-/- ^B cells were incubated with IgM-IC *in vitro*, MZ B cells bound higher levels of IgM-IC than did FO B cells (3 to 5 times as assessed by mean fluorescence intensity), similar to results using B cells from B6 mice.

**Figure 3:**
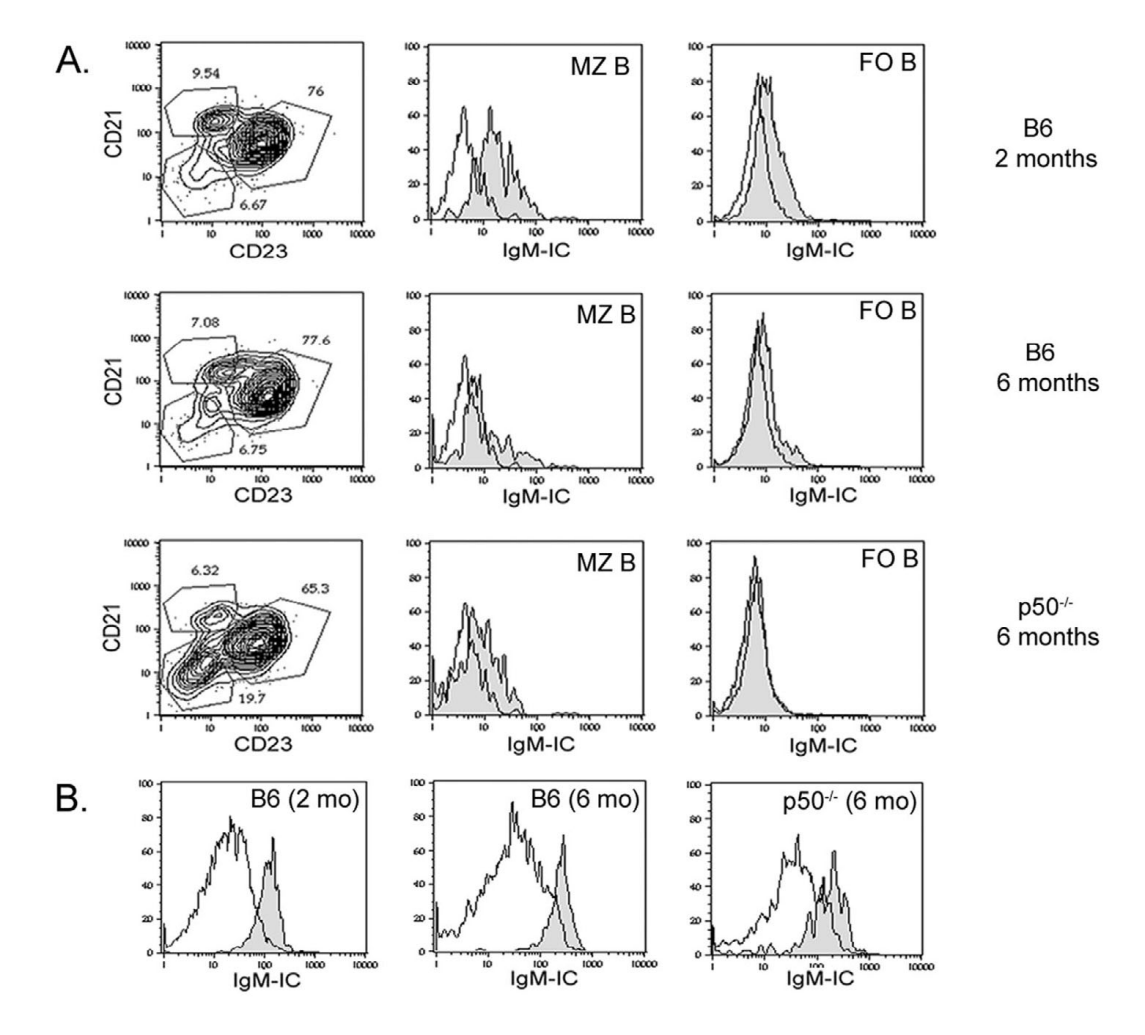
**p50^-/- ^mice accumulate MZ B cells with age that have the capacity to bind high levels of IgM-IC A. **FACS profile of splenocytes from 2 and 6 month old B6 and 6 month p50^-/- ^mice reveal the presence of normal numbers of MZ B cells in spleens of the p50^-/- ^mice. Histograms illustrate binding of IgM-IC to p50^-/- ^MZ B cells as described in Figure 2A. **B. ***In vitro *IgM-IC binding assay. Binding to MZ B cells (shaded profiles); binding to FO B cells (clear profiles).

Note that the spleens of p50^-/- ^mice contain an increased number of CD21^lo^CD23^lo ^B cells (Fig. 2A; see also Fig. 3A). While we have not explored the identity of these cells in detail. they are likely to reflect increased numbers of immature "transitional" B cell subsets, since the number of AA4.1^+ ^B cells [22] in the spleens of these mice are increased (up to 2-fold) over those in age-matched control B6 mice. As expected from their low levels of CD21, however, this population of cells does not bind IgM-IC (data not shown).

MZ B cells can be distinguished as IgM^hi ^B cells positioned outside of the marginal sinus. To determine whether the MZ B cells in p50^-/- ^mice were positioned correctly around the marginal sinus of the spleen, immunofluorescence (IF) was performed on spleen sections using anti-IgM and anti-MOMA-1, which stains marginal metallophilic macrophages and delineates the MZ [23]. In control B6 mice, IgM^hi ^cells (in red) were present in the MZ, outside of the MOMA-1^+ ^cells (Figure 2C). IgM^hi ^MZ B cells were also found outside of the MOMA-1 ring in p50^-/- ^mice, but in reduced numbers, as expected from the drastic reductions in MZ B cells in these mice (Figure 2A). Taken together, these data indicate that p50^-/- ^mice contain small numbers of MZ B cells that are correctly positioned around the marginal sinus, where they can encounter blood-borne IgM-IC and transport them into splenic follicles for deposition onto FDCs.

### Accumulation of MZ B cells with age in p50-deficient mice

**Figure 4:**
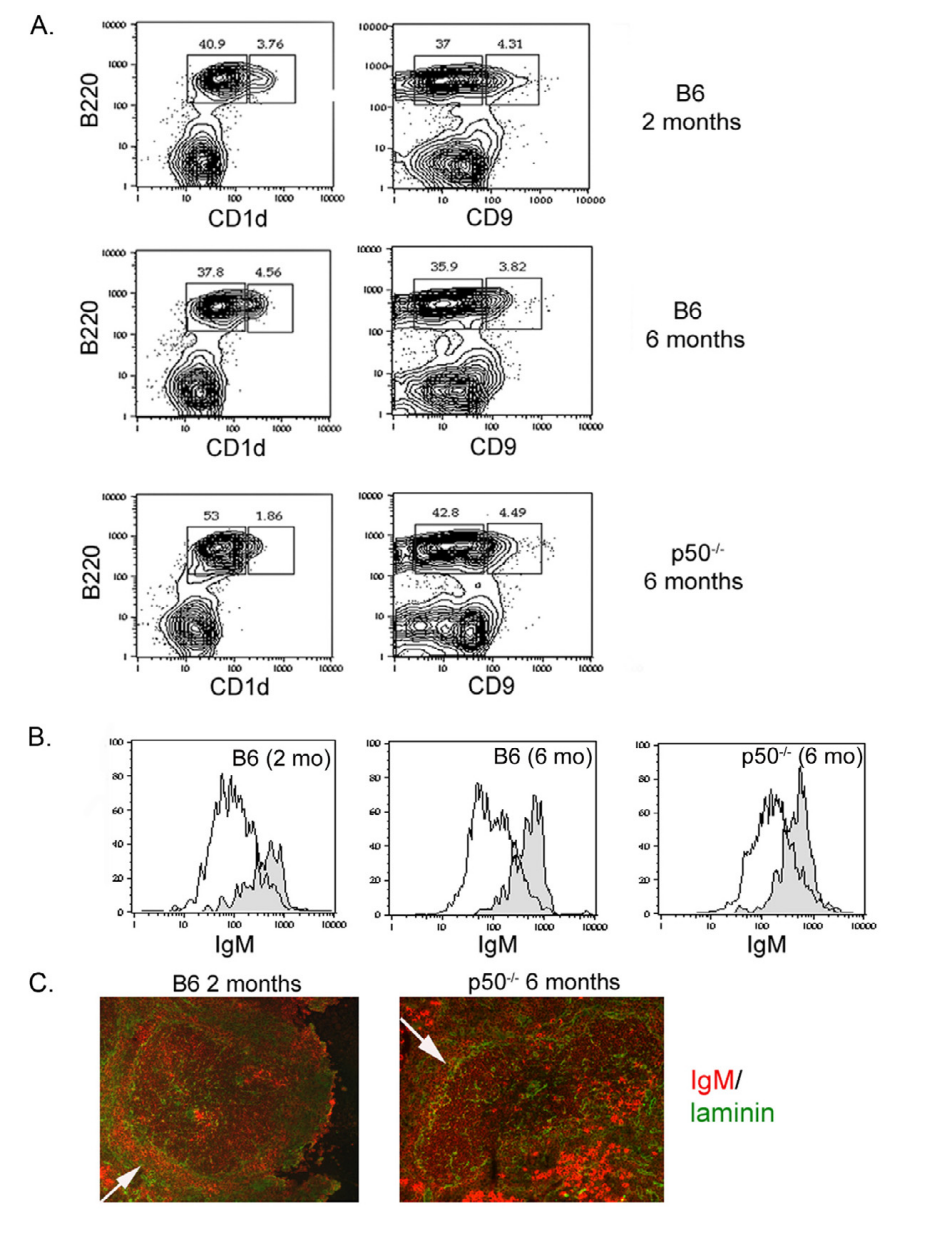
**Phenotypic and histological analysis of MZ B cells in p50^-/- ^mice A. **FACS profile of splenocytes from 2 and 6 month old B6 and 6 month old p50^-/- ^mice revealed by B220/CD1d and B220/CD9 profiles. **B. **FACS histograms showing IgM levels on MZ and FO B cells (shaded and clear regions, respectively) as determined by CD21/23 profiles. Cells present in the MZ B cell gate in p50^-/- ^mice express higher levels of IgM compared with FO B cells, similar to B6 mice. **C. **IF reveals well demarcated IgM^hi ^cells (in red) in the MZ outside of the laminin staining (which identifies the marginal sinus; in green) in 6 month p50^-/- ^mice.

**Figure 5:**
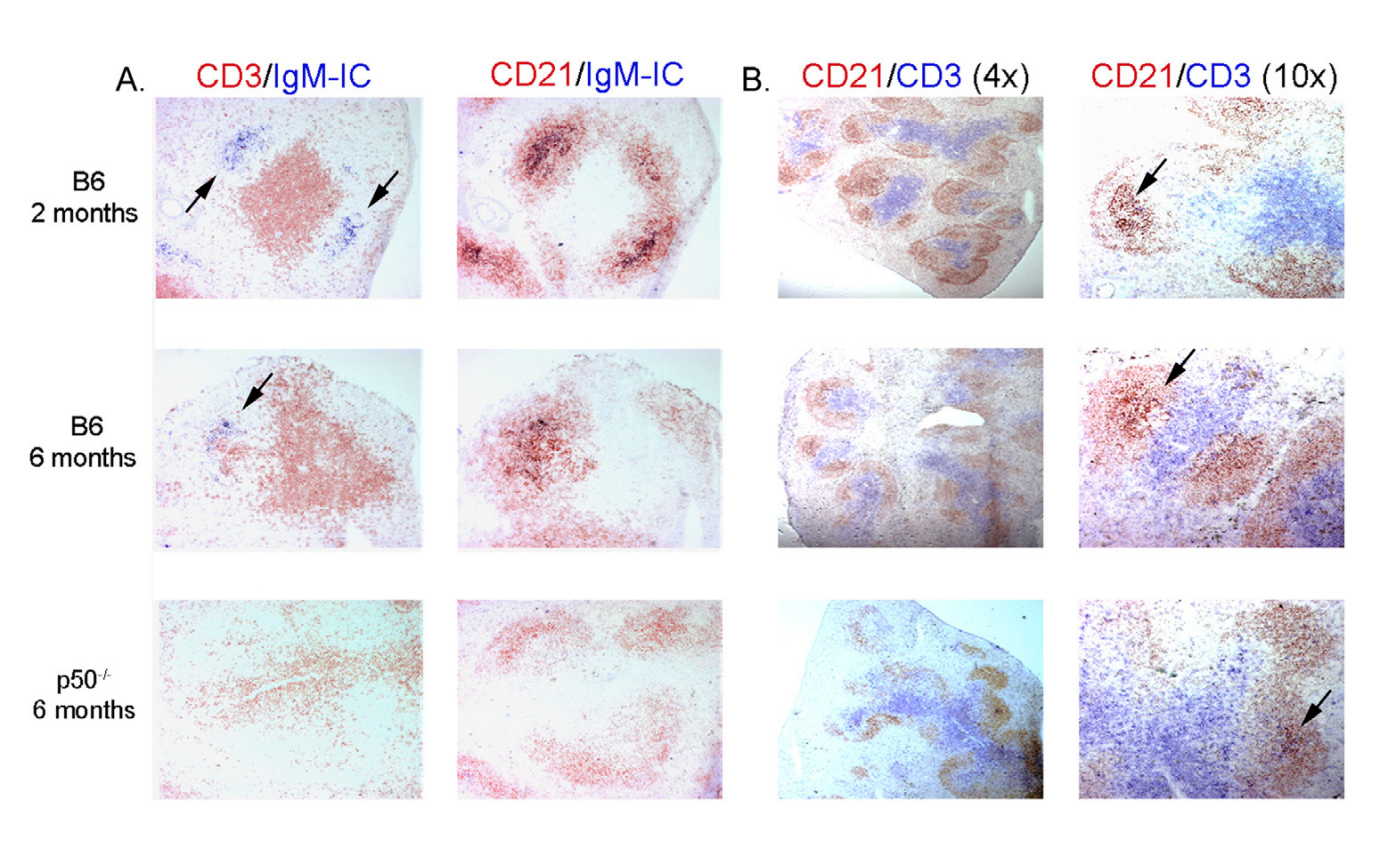
**Deteriorated FDCs in aged p50^-/- ^mice correlates with reduced IgM-IC localization A. **2 month and 6 month old B6 mice and 6 month old p50^-/- ^mice were injected with IgM-IC and analyzed by IH for IgM-IC localization 16 h later. IgM-IC were present on FDCs in 2 and 6 month B6 mice (see arrows), but were not deposited onto FDCs in p50^-/- ^mice. **B. **Intense CD21 staining (designated by arrows) was less visible in the spleens of p50^-/- ^mice, indicating that FDC networks were poorly developed in these mice. This suggests that reduced IgM-IC localization may be attributed to the loss of functional FDC networks in p50^-/- ^mice. The magnifications noted refer to the objective used, not the absolute magnification.

When older p50^-/- ^mice were analyzed for the presence of MZ B cells, increased frequencies of MZ B cells were detected. At 4 months of age, some p50^-/- ^mice contained increased numbers of MZ B cells, whereas others did not, suggesting that these cells might be emerging around this time period (data not shown). By 6 months of age, a phenotypically distinct MZ B cell population was detected in all of the more than 10 mice analyzed, as determined by the CD21/23 profile. In these mice, MZ B cells were present at frequencies comparable to those found in B6 mice (Figure 3A, left panels), although the total number of splenic B cells did not appear to change. At all ages tested, p50^-/- ^and B6 mice had similar frequencies of T and B cells in their spleens, and their spleen sizes were also similar (unpublished results), and the cellularity of the spleens (as determined by IH; see for example Fig. 5B) were also similar, indicating that the increased number of MZ B cells was selective for this subset of cells. To confirm the phenotypic identity of the CD21^hi^/CD23^lo ^B cells as MZ B cells, the expression of other markers of MZ B cells, including CD1d [20] and CD9 [24], was assessed. As shown in Figure 4A, populations of B220^+ ^B cells with CD1d^hi ^and CD9^hi ^phenotypes were present in p50^-/- ^mice, consistent with the presence of MZ B cells. The frequencies of these cells were similar to those in aged-matched control B6 mice. We also assessed the levels of IgM on the CD21^hi^CD23^lo ^cells, since MZ B cells express higher levels of IgM compared with FO B cells [25]. MZ B cells from p50^-/- ^mice clearly expressed higher levels of IgM than on FO B cells from these same mice, and at levels similar to those in control mice (Figure 4B). These B cells were also positioned within the MZ in p50^-/- ^mice (Figure 4C), as indicated by the presence of IgM^+ ^cells outside of the marginal sinus as revealed by laminin staining [26].

As further evidence that the accumulating cells were functional MZ B cells, cells were tested for their capacity to bind IgM-IC *in vivo *and *in vitro*. MZ B cells from 2 and 6 month old B6 mice or from 6 month old p50^-/- ^mice had high levels of IgM-IC bound following i.v. injection, whereas FO B cells bound little or no IgM-IC (Figure 3A). An *in vitro *binding assay also demonstrated the capacity of the MZ B cells from p50^-/- ^mice to bind high levels of IgM-IC compared to FO B cells (Figure 3B). Taken together, the data demonstrate that while few MZ B cells are present in young p50^-/- ^mice, they do develop and accumulate with age, such that by 6 months they have reached normal levels and can functionally bind IgM-IC at high levels, as would be expected from their increased expression of CR1/2 (CD21/35).

### Accelerated loss of FDC networks in p50-deficient mice

In an effort to determine whether the repopulation of older p50^-/- ^mice with MZ B cells enabled efficient IgM-IC trapping, mice were injected with IgM-IC and analyzed for FDC deposition. Despite the normal levels and positioning of MZ B cells, no IgM-IC deposition was observed in the spleens of 6 month old p50^-/- ^mice (Figure 5A). The failure of the IgM-IC to localize in the follicles of these mice could be due to the absence of FDCs. However, as shown in Figure 5B, FDCs were present in these mice, as assessed by CD21 staining. However, they appeared to be much smaller and the dendrites less pronounced than in age-matched B6 mice, as evident from the diminished area of intense CD21 staining (Figure 5B, right panels; see arrows). In addition, the number of follicles with intense CD21 staining was reduced in the 6 month old p50^-/- ^mice compared with age matched B6 mice (Figure 5B, 4× view in left panels). This suggests that the ability of FDCs from p50^-/- ^mice to retain complement fragments might be diminished at 6 months of age. Interestingly, 6 month old B6 mice also exhibited some reduced capacity to trap IgM-IC (Figure 5A) and slightly reduced CD21 staining compared with younger mice (Figure 5B), suggesting a loss of FDC function may also be occurring naturally with age, as previously observed [27]. Consistent with this, FDCs in 6 month old p50^-/- ^and B6 mice could not be detected by FDC-M2 staining (data not shown), in contrast with younger mice (Figure 1). FDC-M2 is a FDC specific antibody [28] that detects the complement component C4 on FDCs [29]. The data indicate the loss of FDC function may be accelerated in mice lacking p50. Together, the data imply that the lack of FDC localization of IgM-IC in 6 month old p50-deficient mice is not due to functional inactivity of the MZ B cells that have accumulated in these mice, but reflects a loss of FDCs and/or FDC function by this age.

## Discussion

Our data demonstrate that while NF-κB p50^-/- ^mice are clearly deficient in MZ B cells for the first 2 months of life, MZ B cells appear with age, such that by 6 months a normal splenic MZ B cell compartment has been reconstituted. There are at least four distinct but not mutually exclusive mechanisms that could account for the late appearance of cells within the MZ of p50^-/- ^mice. First, these B cells could be memory B cells, which have been reported to accumulate in the MZ [30]. While we cannot formally rule out this possibility, B cells from p50^-/- ^mice respond poorly to antigenic challenge, especially T-dependent antigens [16]. Consequently, it is unlikely that memory B cells would be generated in these mice, irrespective of their age. Alternatively, the emergence of MZ B cells in p50^-/- ^mice could represent the accumulation of these cells through homeostatic proliferation, in which cells proliferate to fill niches in response to deficits in specific subsets of cells [31]. While splenic B cells from p50^-/- ^mice will undergo homeostatic proliferation as a consequence of their transfer into SCID recipients [32], the ability of the few MZ B cells in the p50^-/- ^donor spleen to expand following transfer was not examined [32]. Nevertheless, we view it as unlikely that homeostatic proliferation accounts for the accumulation of MZ B cells in the spleens of older p50^-/- ^mice, since the accumulation of cells brought on by homeostatic proliferation is generally observed over a much shorter period of time, days to a couple of weeks, rather than the months required to reconstitute MZ B cells in p50^-/- ^mice. A third possibility is that a NF-κB p50-dependent signal transduction pathway that activates a developmentally required transcription program in MZ B cells or their precursors is overcome by compensatory mechanisms with aging. NF-κB p50 is involved in a number of pathways crucial for B cell development and maturation, including B cell receptor (BCR) signalling and responses to members of the TNF family [33-35] Signal strength through the BCR has been proposed to be an important determinant of B cell lineage fate decisions, including the MZ B cell pool [36,37] BAFF signalling is also important for the selection of B cells into the MZ B cell pool [38], while an intact LT-β R signalling pathway is required for the proper positioning of these cells [39]. The accumulation of MZ B cells in older p50^-/- ^mice could reflect compensatory mechanisms in one or more of these signal transduction pathways important for the emergence or maintenance of MZ B cells. A fourth possibility is that the small numbers of MZ B cells that successfully emerge in p50^-/- ^mice survive for extended periods of time, resulting in the slow accumulation of this subset of cells with age. Hao and Rajewsky [40] have demonstrated that the half-life of MZ B cells is greater than that of FO B cells. Thus, a simple explanation for the accumulation of MZ B cells in p50^-/- ^mice with age is that these cells preferentially survive, and thus their frequency relative to the FO B cell pool and other B cells in the spleen increases slowly with age.

B cells from p50^-/- ^mice are compromised in their ability to respond to specific antigen challenge, and are completely unresponsive to mitogenic substances such as LPS [16]. However, our data suggests that MZ B cells from p50^-/- ^mice display some functionality, including the binding, transport and deposition of IgM-IC onto FDCs, a function unique to MZ B cells [15]. Like IgM-containing T-dependent immune complexes, MZ B cells also bind T-independent type 2 (TI-2) antigens in a complement and CR1/2-dependent manner [41]. Despite the fact that p50^-/- ^mice are widely considered to be devoid of MZ B cells, the presence of small numbers of these B cells in p50^-/- ^mice were in fact noted in the initial studies of Pillai and colleagues, and these appeared to bind low levels of TI-2 antigens [17]. However, further analysis of the consequences of this binding, such as the transfer of immune complexes to FDCs, was not evaluated. The results of the current study not only extend these previous studies, but demonstrate that one consequence of the small amount of IgM-IC binding manifests itself by the limited, but none-the-less detectable, deposition of these complexes onto FDCs. This suggests that this function of MZ B cells is completely independent of the activation of NF-κB, at least those complexes containing p50. These results extend other evidence suggesting that the activation of MZ B cells through engagement of the BCR may not be required for transport and deposition of IgM-IC, since MZ B cells from CD19-deficient mice, which also have deficits in MZ B cells, are also functional in this assay [15].

Together, these results raise the question of whether MZ B cells require activating signals to migrate into B cell follicles following IgM-IC binding to CR1/2. MZ B cell migration can be detected in 1 h or less after the binding of IgM-IC [15]. The binding of IgM-IC to these cells may interfere with one or more of the mechanisms proposed to play a role in MZ B cell positioning, including retention by macrophages within the MZ [42], integrin-mediated retention [39], or responsiveness to lysophospholipids in the blood [43]. Once released, the MZ B cells would be expected to migrate into follicles in response to chemoattractants such as BLC [44]. These considerations suggest that follicular migration of MZ B cells may be the "default" pathway, whereas BCR engagement, which is known to direct MZ B cells not into the follicles but to the T-B interface [45-47], actively alters the fate of antigen specific MZ cells away from follicular migration.

Despite the increase in frequency (and numbers) of MZ B cells in p50^-/- ^mice, the deposition of IgM-IC within the follicles does not improve with age, but declines severely. This appears to be due to a decrease in function of FDCs, which are fewer in number and are less pronounced, as evidenced by CD21 staining (Figure 5). Thus, the decreased ability of FDCs to retain complement containing immune complexes observed in old mice [27], appear to emerge with accelerated kinetics in p50^-/- ^mice.

NF-κB p50 is only one of a growing number of genes, including a number of transcription factors, whose expression influences the development of MZ B cells (reviewed in [48]), highlighting the complex interactions that must converge to generate this B cell compartment. The fact that the requirements for individual proteins, such as p50, could change with age suggests that compensatory mechanisms exist which can overcome cellular defects, and/or that more than one pathway to generate these cells may exist.

## Conclusion

These experiments demonstrate the profound influence age can exert on cellular populations in genetically modified mice. The appearance of large numbers of MZ B cells in 6 month old p50^-/- ^mice suggests that compensatory mechanisms overcome the deficits caused by p50-deficiency. Both in young and old p50^-/- ^mice, these MZ B cells appear to retain some functionality, as assessed by their ability to bind, transport and deposit IgM-IC onto FDCs. On the other hand, FDCs are unable to maintain their functional capacity to trap IgM-IC in aged p50^-/- ^mice indicating that, while MZ B cells recover with age, other immune defects become more pronounced as a result of deficiency in this member of the NF-κB family of transcription factors.

## Methods

### Mice

C57BL/6J and NF-κB p50 deficient (B6;129P2-Nfkb1^tm1Bal^) [16] mice were purchased from Jackson Laboratories (Bar Harbor, ME). Mice were maintained in the Laboratory Animal Sciences Center, Boston University Medical Center. All studies were reviewed by an Institutional Animal Care and Use Committee and carried out in accordance with established guidelines according to National Institutes of Health. Mice of both sexes were used at 2 to 6 months of age as described.

### Preparation and injection of IgM-containing immune complexes (IgM-IC)

Mice were injected i.v. with a mixture of 15 to 20 μg each of NP_12_-BSA-biotin (Biosearch Technologies, Novato, CA) and affinity purified pentameric IgM anti-NP monoclonal antibody (B1-8) exactly as previously described [7,15].

### *In vitro *binding assay

*In vitro *assays to assess the binding of IgM-IC to MZ B cells were performed as previously described [15]. Briefly, IgM-IC were formed by mixing 1 μg each of NP-BSA-biotin and B1-8 IgM in RPMI in the presence of 10% fresh mouse serum as a source of complement and incubated for 30 minutes at 37°C. Splenocytes (10^7^) were resuspended in the IgM-IC and incubated at 37°C for an additional 30 minutes. Cells were washed and analyzed by flow cytometry.

### Immunohistochemistry (IH)

Staining of spleen sections was performed exactly as previously described [7,15]. Briefly, spleens were removed and snap frozen in O.C.T. Compound (Tissue Tek, Torrance, CA) and stored at -80°C. 6 μm sections were cut (IEC Microtome, Needham, MA) and fixed in acetone at -20°C for 10 min. Sections were incubated with 0.3% H_2_O_2_/PBS for 5 min and blocked with 3% normal rabbit or rat serum. All antibodies were diluted in 5% BSA in PBS. Primary antibodies used included: anti-CD3-FITC (1/200), anti-CD3-biotin (1/200), anti-CD21/35-FITC (1/100), (all from BD Biosciences, San Diego), ER-TR9 (1/200; BMA Biomedical, Augst, Switzerland), and anti-FDC-M2 (1/200; Immunokontact, Frankfurt, Germany). Secondary antibodies included anti-FITC-HRP (1/100; Roche Molecular Biochemicals, Indianapolis, IN), goat anti-rat IgG-HRP (1/500; Jackson Immunoresearch, West Grove, PA), and goat anti-rat IgM (Fab')_2_-HRP (1/1000; Jackson Immunoresearch). NP_12_-BSA-biotin and anti-CD3-biotin was visualized by alkaline phosphatase (AP)-streptavidin (1/400; Molecular Probes, Eugene, OR) and developed with napthol AS-MX phosphate/Fast blue BB base (Sigma, St. Louis, MO). Visualization of antibody stains was accomplished using horse radish peroxidase (HRP) conjugated secondary antibodies developed with 3-amino-9-ethylcarbazole (Sigma) and H_2_0_2_. After staining, sections were covered with crystal mount (Biomedia, Foster City, CA) before examining by digital microscopy (Zeiss, Axiovert 200 M, Oberkochen, Germany). Unless otherwise noted, all images were obtained using a 10× objective (initial magnification of 100×) unless otherwise noted (see Fig. 4).

### Immunofluorescence (IF)

Sections were initially treated as for IH. Antibodies utilized included anti-MOMA-1 (1/25; Serotec, Raleigh NC) visualized with a goat anti-rat IgG-AMCA (1/100; Jackson Immunoresearch), anti-IgM-Texas Red (1/100; BD Biosciences), and anti-laminin (1/500; Sigma) visualized with an anti-rabbit IgG-FITC (1/100; Jackson Immunoresearch). All sections were covered with Fluoromount G (Jackson Immunoresearch) and a cover slip before examining by digital microscopy (Zeiss, Axiovert 200 M, Oberkochen, Germany).

### Flow cytometry

Splenocytes were isolated at the indicated times and stained as follows. The lymphocyte gate was utilized for all analysis. Cells were analyzed using a FACScan (Becton Dickinson, Franklin Lakes, NJ) and FlowJo software (Tree Star, Ashland, OR). Antibodies utilized were anti-B220-FITC (1/200), anti-B220-biotin (1/200), anti-B220-PE (1/200), anti-CD21/35-FITC (1/200), anti-CD23-PE (1/200), and anti-CD1d-FITC or PE (1/100) (BD Biosciences). Streptavidin-Cy5 (1/200; BD Biosciences) was used to identify the NP antigen (NP_12_-BSA-biotin) associated with lymphocytes, as well as to visualize biotin-labelled antibodies.

## Authors' contributions

A.R.F. performed the experiments reported in this manuscript and participated in the data interpretation and analysis. R.B.C. originally conceived of the study, participated in data interpretation and analysis, and takes overall responsibility for the conduct of the studies. Both authors have read and approve of the final manuscript.

## References

[B1] Henry C, Jerne NK (1968). Competition of 19S and 7S antigen receptors in the regulation of the primary immune response. J Exp Med.

[B2] Dennert G (1971). The mechanism of antibody-induced stimulation and inhibition of the immune response. J Immunol.

[B3] Heyman B, Andrighetto G, Wigzell H (1982). Antigen-dependent IgM-mediated enhancement of the sheep erythrocyte response in mice. Evidence for induction of B cells with specificities other than that of the injected antibodies. J Exp Med.

[B4] Harte PG, Cooke A, Playfair JHL (1983). Specific monoclonal IgM is a potent adjuvant in murine malaria vaccination. Nature.

[B5] Heyman B, Wigzell H (1985). Specific IgM enhances and IgG inhibits the induction of immunological memory in mice. Scand J Immunol.

[B6] Basalp A, Cirakoglu B, Bermek E (2000). Enhancement of the immune response to hepatitis B virus vaccine by antigen specific IgM. Immunol Lett.

[B7] Youd ME, Ferguson AR, Corley RB (2002). Synergistic roles of IgM and complement in antigen trapping and follicular localization. Eur J Immunol.

[B8] Heyman B, Pilström L, Shulman MJ (1988). Complement activation is required for IgM-mediated enhancement of the antibody response. J Exp Med.

[B9] Applequist SE, Dahlstrom J, Jiang N, Molina H, Heyman B (2000). Antibody production in mice deficient for complement receptors 1 and 2 can be induced by IgG/Ag and IgE/Ag, but not IgM/Ag complexes. J Immunol.

[B10] Boes M, Esau C, Fischer MB, Schmidt T, Carroll M, Chen J (1998). Enhanced B-1 cell development, but impaired IgG antibody responses in mice deficient in s ecreted IgM. J Immunol.

[B11] Boes M, Prodeus AP, Schmidt T, Carroll MC, Chen J (1998). A critical role of natural immunoglobulin M in immediate defense against systemic bacterial infection. J Exp Med.

[B12] Ehrenstein MR, O'Keefe TL, Davies SL, Neuberger MS (1998). Targeted gene disruption reveals a role for natural secretory IgM in the maturation of the primary immune response. Proc Natl Acad Sci USA.

[B13] Baumgarth N, Herman OC, Jager GC, Brown LE, Herzenberg LA, Chen J (2000). B-1 and B-2 cell-derived immunoglobulin M antibodies are nonredundant components of the protective response to influenza virus infection. J Exp Med.

[B14] Ochsenbein AF, Fehr T, Lutz C, Suter M, Brombacher F, Hengartner H, Zinkernagel RM (1999). Control of early viral and bacterial distribution and disease by natural antibodies. Science.

[B15] Ferguson AR, Youd ME, Corley RB (2004). Marginal zone B cells transport and deposit IgM-containing immune complexes onto follicular dendritic cells. Int Immunol.

[B16] Sha WC, Liou HC, Tuomanen EI, Baltimore D (1995). Targeted disruption of the p50 subunit of NF-κB leads to multifocal defects in immune responses. Cell.

[B17] Cariappa A, Liou HC, Horwitz BH, Pillai S (2000). Nuclear factor κB is required for the development of marginal zone B lymphocytes. J Exp Med.

[B18] Oliver AM, Martin F, Gartland GL, Carter RH, Kearney JF (1997). Marginal zone B cells exhibit unique activation, proliferative and immunoglobulin secretory responses. Eur J Immunol.

[B19] Takahashi K, Kozono Y, Waldschmidt TJ, Berthiaume D, Quigg RJ, Baron A, Holers VM (1997). Mouse complement receptors type 1 (CR1;CD35) and type 2 (CR2;CD21): expression on normal B cell subpopulations and decreased levels during the development of autoimmunity in MRL/lpr mice. J Immunol.

[B20] Amano M, Baumgarth N, Dick MD, Brossay L, Kronenberg M, Herzenberg LA, Strober S (1998). CD1 expression defines subsets of follicular and marginal zone B cells in the spleen: beta 2-microglobulin-dependent and independent forms. J Immunol.

[B21] Makowska A, Faizunnessa NN, Anderson P, Midtvedt T, Cardell S (1999). CD1high B cells: a population of mixed origin. Eur J Immunol.

[B22] Allman D, Lindsley RC, DeMuth W, Rudd K, Shinton SA, Hardy RR (2001). Resolution of three nonproliferative immature splenic B cell subsets reveals multiple selection points during peripheral B cell maturation. J Immunol.

[B23] Kraal G, Janse M (1986). Marginal metallophilic cells of the mouse spleen identified by a monoclonal antibody. Immunology.

[B24] Won WJ, Kearney JF (2002). CD9 is a unique marker for marginal zone B cells, B1 cells, and plasma cells in mice. J Immunol.

[B25] Kumararatne DS, MacLennan IC (1981). Cells of the marginal zone of the spleen are lymphocytes derived from recirculating precursors. Eur J Immunol.

[B26] Wang SL, Kutsche M, DiSciullo G, Schachner M, Bogen SA (2000). Selective malformation of the splenic white pulp border in L1-deficient mice. J Immunol.

[B27] Aydar Y, Balogh P, Tew JG, Szakal AK (2004). Follicular dendritic cells in aging, a "bottle-neck" in the humoral immune response. Ageing Res Rev.

[B28] Kosco-Vilbois MH, Zentgraf H, Gerdes J, Bonnefoy JY (1997). To 'B' or not to 'B' a germinal center?. Immunol Today.

[B29] Taylor PR, Pickering MC, Kosco-Vilbois MH, Walport MJ, Botto M, Gordon S, Martinez-Pomares L (2002). The follicular dendritic cell restricted epitope, FDC-M2, is complement C4; localization of immune complexes in mouse tissues. Eur J Immunol.

[B30] Liu YJ, Oldfield S, MacLennan IC (1988). Memory B cells in T cell-dependent antibody responses colonize the splenic marginal zones. Eur J Immunol.

[B31] Surh CD, Sprent J (2002). Regulation of naive and memory T-cell homeostasis. Microbes Infect.

[B32] Cabatingan MS, Schmidt MR, Sen R, Woodland RT (2002). Naive B lymphocytes undergo homeostatic proliferation in response to B cell deficit. J Immunol.

[B33] Gold MR, Ingham RJ, McLeod SJ, Christian SL, Scheid MP, Duronio V, Santos L, Matsuuchi L (2000). Targets of B-cell antigen receptor signaling: the phosphatidylinositol 3-kinase/Akt/glycogen synthase kinase-3 signaling pathway and the Rap1 GTPase. Immunol Rev.

[B34] Gugasyan R, Grumont R, Grossmann M, Nakamura Y, Pohl T, Nesic D, Gerondakis S (2000). Rel/NF-κB transcription factors: key mediators of B-cell activation. Immunol Rev.

[B35] Aggarwal BB (2003). Signalling pathways of the TNF superfamily: a double-edged sword. Nat Rev Immunol.

[B36] Cariappa A, Tang M, Parng C, Nebelitskiy E, Carroll M, Georgopoulos K, Pillai S (2001). The follicular versus marginal zone B lymphocyte cell fate decision is regulated by Aiolos, Btk, and CD21. Immunity.

[B37] Casola S, Otipoby KL, Alimzhanov M, Humme S, Uyttersprot N, Kutok JL, Carroll MC, Rajewsky K (2004). B cell receptor signal strength determines B cell fate. Nat Immunol.

[B38] Mackay F, Woodcock SA, Lawton P, Ambrose C, Baetscher M, Schneider P, Tschopp J, Browning JL (1999). Mice transgenic for BAFF develop lymphocytic disorders along with autoimmune manifestations. J Exp Med.

[B39] Lu TT, Cyster JG (2002). Integrin-mediated long-term B cell retention in the splenic marginal zone. Science.

[B40] Hao Z, Rajewsky K (2001). Homeostasis of peripheral B cells in the absence of B cell influx from the bone marrow. J Exp Med.

[B41] Guinamard R, Okigaki M, Schlessinger J, Ravetch JV (2000). Absence of marginal zone B cells in Pyk-2-deficient mice defines their role in the humoral response. Nat Immunol.

[B42] Karlsson MC, Guinamard R, Bolland S, Sankala M, Steinman RM, Ravetch JV (2003). Macrophages control the retention and trafficking of B lymphocytes in the splenic marginal zone. J Exp Med.

[B43] Cinamon G, Matloubian M, Lesneski MJ, Xu Y, Low C, Lu T, Proia RL, Cyster JG (2004). Sphingosine 1-phosphate receptor 1 promotes B cell localization in the splenic marginal zone. Nat Immunol.

[B44] Cyster JG, Ansel KM, Reif K, Ekland EH, Hyman PL, Tang HL, Luther SA, Ngo VN (2000). Follicular stromal cells and lymphocyte homing to follicles. Immunol Rev.

[B45] Liu YJ, Zhang J, Lane PJ, Chan EY, MacLennan IC (1991). Sites of specific B cell activation in primary and secondary responses to T cell-dependent and T cell-independent antigens. Eur J Immunol.

[B46] Martin F, Oliver AM, Kearney JF (2001). Marginal zone and B1 B cells unite in the early response against T-independent blood-borne particulate antigens. Immunity.

[B47] Song H, Cerny J (2003). Functional heterogeneity of marginal zone B cells revealed by their ability to generate both early antibody-forming cells and germinal centers with hypermutation and memory in response to a T-dependent antigen. J Exp Med.

[B48] Martin F, Kearney JF (2002). Marginal-zone B cells. Nat Rev Immunol.

